# Artificial intelligence attitudes and resistance to use robo-advisors: exploring investor reluctance toward cognitive financial systems

**DOI:** 10.3389/frai.2025.1623534

**Published:** 2025-09-17

**Authors:** Balraj Verma, Mike Schulze, Divya Goswami, Kamal Upreti

**Affiliations:** ^1^Chitkara Business School, Chitkara University, Punjab, India; ^2^CBS International Business School, CBS University of Applied Sciences, Mainz, Germany; ^3^Department of Computer Science, Christ University, Delhi, India

**Keywords:** attitude toward AI, robo-advisors, artificial intelligence, resistance, FRA, cognitive financial systems

## Abstract

**Introduction:**

The study investigates resistance towards Financial Robo-Advisors (FRAs) among retail investors in India, grounded in innovation resistance theory. The study examines the impact of functional barriers and psychological barriers on resistance to FRAs, while considering user’s attitudes towards Artificial Intelligence (AI) as a moderator. It further evaluate the influence of such resistance on users’ intentions to use and recommend FRAs.

**Methods:**

Utilizing purposive sampling data was collected from 409 investors and further analyzed using structural equation modelling.

**Results:**

The findings revealed that all barriers under study, expect value barrier, substantially derive resistance towards robo-advisors, with inertia being the strongest determinant. Further, this resistance impedes both the intention to use FRAs and to recommend them. Moderation analysis results finds that users’ attitude towards AI significantly weakens the influence of inertia, overconfidence bias and data privacy risk on resistance, with no such impact on other relationships.

**Discussion:**

Overall, the study enriches IRT in Fintech context and provides theoretical and practical insights to enhance FRAs adoption in emerging markets.

## 1 Introduction

In the era defined by rapid technology transformations, the financial service industry has observed paradigm change driven with advancements with artificial intelligence (AI), machine learning, and digitalization. AI-based technology has realized its potential in the banking and financial services sector, compelling the rise of Fintech services (Josyula and Expert, 2021). Among all these innovations, financial robo-advisors (FRAs, hereafter) have emerged a disruptive force, providing algorithm-driven financial advice at affordable prices as compared to conventional advisors. These digital advisory services provide 24/7 support services with ease of accessibility with limited resources (Bhatia et al., 2021). A robo-advisor guides investors through a self-assessment process by using algorithms for making goal-based financial decisions. In addition, these digital platforms provide highly efficient, accessible, and cost-effective assistance to manage their investments at any time. By using user-friendly interfaces and instructional material, they help investors navigate the complexity of financial assets in response to the market fluctuations (Salo and Haapio, 2017). Moreover, when compared with the traditional advisors, they charge fees frequently for any extra services that may or may not be relevant for the investor’s needs. Conversely, FRAs provide a more inclusive and cost-effective option making financial planning services available to a wide range of investors. According to “Robo-advisors-Worldwide” report by Statista (2024), the average assets under management in the market of robo-advisors are predicted to US$61.9 k in 2025 globally. The potential of financial robo-advisory services to transform wealth management services is undeniable.

Despite their growing recognition, adoption of these robo-advisory services remains suboptimal (Jung et al., 2018; Luo et al., 2024; Lee et al., 2025), specifically investors’ reluctance to use these platforms. There is a candid need to motivate investors and reduce their unwillingness to use robo-advisory services, which seems to be the most vital hurdle that service providers must overcome. It has been seen that many of the investors prefer sticking to their status quo and look forward to get advisory services offered by the human or conventional advisors. This could be due to the psychological barriers such as distrust in the technology, emotional discomfort, negative image of these digital platforms, and functional barriers such as complexity, privacy, and security issues, that are holding back the investors to accept these AI-backed financial services thus restricting the diffusion.

Developing a new understanding of the factors that leads to psychological discomfort or reluctance to use FRAs becomes necessary for their large-scale acceptance. The extant literature has primarily focused on the design of robo-advisors (Jung et al., 2018), behavioral biases (Raheja and Dhiman. 2019), anthropomorphism in robo-advisors (Goswami et al., 2025; Adam et al., 2019), building trust in robo-advisors (Nourallah et al., 2022), and investors willingness (Luo et al., 2024). Previous research has also thrown light on investment strategies (Alsabah et al., 2021) and risks and returns (Oehler and Horn, 2024). However, these studies frameworks ignore vital psychological elements that influence user behavior, deriving resistance to use FRAs. The study seeks to investigate the barriers that are responsible for the user’s unwillingness to use FRAs. Sinclair et al. (2015), negative emotions have a greater impact on the decision-making by the individuals that the positive emotions due to their tendency to focus on negativity than positivity. Although, very little effort has been made to understand the negative or resisting nature of investors toward FRAs.

Although the resistance by the users has a crucial role toward the adoption of the FRAs, it becomes equally important to understand how these resistances further shapes the behavioral outcomes, i.e., intention to use and intention to recommend FRAs. The previous literature on technology acceptance has also stated that the resistance does not operate alone but influences significantly the behavioral intentions (Talwar et al., 2020; Lee and Kim, 2022). In context to robo-advisory services, the resistance is simply rooted in both psychological and functional barriers which not only impedes the actual usage but also individual’s intentions to recommend it to others (Ram and Sheth, 1989). Furthermore, analyzing both the user’s intentions and intentions to recommend becomes vital for comprehending the broader impact of resistance toward the technology by these individuals. In addition, the users’ intentions to recommend FRAs which is a key post-adoption behavior would also serve as a proxy for user satisfaction in the robo-based financial services. Therefore, including both of the intentions offers a more nuanced understanding of how the resistance affects the users’ intention to adopt and to recommend the FRAs.

Following the section the rest of the study is structured as follows: (1) Introduction; (2) In-depth review of literature; (3) Developing hypotheses and the conceptual framework; (4) Methodology and analytical approach; (5) Data analysis; (6) Discussions and findings; (7) Conclusion with implications, limitations, and future research work.

## 2 Theoretical underpinning

FRAs are digital platforms that provide automated financial services and portfolio management services to investor (Luo et al., 2024). Robo-advisory services assist in financial planning making it more accessible, easily understandable, cost-effective solutions by offering round the clock support system (Bhatia et al., 2021). Individuals’ decisions in context to their hard-earned money demand for the usage of such AI-based automated services, reflecting technology accessibility and need for financial security and privacy. The existing literature on robo-advisory services has explored many dimensions such as design (Jung et al., 2018), satisfaction (Cheng and Jiang, 2020), trust (Zhang et al., 2021), usefulness (Belanche et al., 2019), and factors influencing adoption intention (Kwon et al., 2022). Furthermore, robo advisory services have expanded into different settings and businesses, including portfolio management and investment (Banerjee, 2025), wealth management (Nguyen et al., 2023), and retirement planning (Chhatwani, 2022).

However, little efforts have been paid toward the darker side of these automated services, namely the phenomena of resistance to the information provided by these FRAs. The study is grounded on the innovation resistance theory framework by Ram and Sheth (1989) that suggests that individuals may be hesitant to show confidence on the technology-based information due to perceived concerns, lack of transparency, and psychological discomfort with technology-based financial decision-making processes. An investor has multiple reasons for resistance such as due to the fear of losing control over his hard-earned money and investments, or distrust in AI-based technology amid market volatilities or may fear of algorithmic errors. The study will significantly contribute toward the literature by using IRT theory to investigate the psychological and behavioral biases that cause resistance by the investors to resist FRAs, despite their present potential advantages.

### 2.1 Innovation resistance theory

Innovation resistance theory (IRT) offers an inclusive framework for understanding the user resistance behavior to innovations (Ram and Sheth, 1989). According to IRT, individuals may resist or feel hesitant to accept innovation that they perceive to be risky, irrelevant, or contradictory to the existing status quo and pre-existing value system (Hew et al., 2019). The resistance by the users plays a crucial role in determining whether the innovation will be a success or failure (Ram and Sheth, 1989; Kaur et al., 2020). An investor’s resistance may vary from active to passive resistance (Chawla et al., 2024a; Chawla et al., 2024b). Active resistance stems from the issues related to innovation’s perceived utility, value, and perceived usefulness, whereas passive resistance stems from the psychological barriers such as traditional ideas and beliefs. IRT’s comprehensive approach makes it idyllic for the evaluation of investors’ resistance to innovation (Chawla et al., 2024a; Chawla et al., 2024b). This IRT framework differs from other frameworks as it primarily focuses on value, tradition, usage, risk, and image (Gupta and Arora, 2017). Unlike the models such as UTAUT and TAM, which have a majority of emphasis on the technology adoption through the constructs of ease of use, perceived usefulness etc., the innovation resistance theory (IRT) provides a better perspective of directly addressing the challenges related to the functional and psychological resistance that impede the technology adoption. The distinction becomes quite significant specifically in the context of FRAs, where the reluctance to adopt them is not only influenced by the performance expectations but also by the inertia, behavioral tendencies, and other perceived uncertainties which are often overlooked by TAM and UTAUT. Similarly, although the behavioral reasoning theory (BRT) also acknowledges the conceptual beliefs and the motivations behind the user decision-making, still it does not specifically dissect the innovation-related factors as the theory of innovation resistance does through its structural approach with special focus on functional and psychological resistance. Although the aim of the present research is to explore the reluctance in both emotional and cognitive responses to AI-based robo-advisors, IRT is theoretically more aligned. This clarification will strengthen the study’s conceptual foundation and also validate the selection of the IRT model in the research study.

The literature indicates an increasing interest in understanding innovation resistance, particularly in context to the digitalized services. Numerous studies have employed IRT theoretical framework such as online gamification (Oktavianus et al., 2017), banking services (Matsuo et al., 2018), online travel (Talwar et al., 2020), mobile banking (Kaur et al., 2020), online communities (Kumar et al., 2025), and online-to-offline (O2O) technology platform (Chawla et al., 2024a; Chawla et al., 2024b).

Unlike other online platforms, robo-advisory services involve sensitive financial data, complex algorithms procedures, and financial implications for the users (D’Acunto and Rossi, 2021). There is also a distinction between the user–advisor interactions, lacking the nuanced knowledge and emotional intelligence associated with the human advisors. The existing studies focus on the technology adoption factors, ignoring the potential resistance factors arising from these unique attributes. As a result, our study applies the IRT framework to investigate the factors influencing the resistance behavior of the investors in the robo-advisory services context. Although the previous research studies focusing on the IRT have largely examined the consumer barriers and the adoption intention relationships such as tourism sector (Ahmad and Rasheed, 2025); context to Mobile Payments Systems (Kaur et al., 2020), considerably very less attention has been paid to user intentions and intention to recommend, i.e., the adoption to recommend. Notably, there is very scarce research on the empirical investigations understanding the resistance barriers that to on the adoption outcomes in the domain of robo-advisors specifically (Cardillo and Chiappini, 2024). Thereby, by inculcating the use intention and the intention to recommend within the IRT framework would bridge this gap and extent the IRT model of innovation resistance toward the adoption patterns. The integration of these constructs would capture both the resistance and the enabling mechanisms, i.e., intention to use and intention to recommend, enhancing the explanatory power of the conceptual model.

### 2.2 Attitude toward AI

Attitude toward AI has emerged as a pivotal construct that has a major role in shaping individuals’ acceptance or reluctance to AI-driven technologies such as financial robo-advisors (FRAs). The theory of innovation resistance underscores that resistance by the individuals is not a sole function of the perceived barriers but it is also impacted by their beliefs and pre-existing beliefs (Ram and Sheth, 1989). A positive attitude toward the AI can work as a cognitive filter that changes the perceptions related to risks and barriers, further transforming the perceived potential threats into opportunities for better decision-making (Araujo et al., 2020). The research on AI highlights that the positive attitudes can alter the belief system, mitigating privacy concerns and can reduce perceived complexities barriers (Oprea et al., 2024), but, at the same time, it is also affected by their pre-existing beliefs and attitudes (Ram and Sheth, 1989).

In addition, the users with a positive stance on AI are less susceptible to psychological biases such as inertia and overconfidence as they feel more comfortable in delegating their tasks to the intelligent systems (Longoni et al., 2019). Overall, a positive attitude toward AI enhances the technological readiness, which further helps in lowering down the psychological differences between the users and technology systems, thus reducing the resistance (Parasuraman, 2000). In context to algorithm-driven FRAs, the decision-making process is data-driven and the attitude toward using AI can realign perceptions, reducing resistance and fostering their engagement. The integration of this moderating variable in the model will extend the IRT by demonstrating how the user orientations toward AI can condition the strengths of both psychological barrier and functional barriers that shape the pathways to the adoption of digital financial systems.

## 3 Research model and hypothesis

### 3.1 Perceived complexity and resistance to FRA

Investor resistance is most often driven by the usage barrier. The barrier arises when the innovation does not align with current workflows, procedures, and habits to hold new systems for their advantage. In other words, the sophisticated algorithm-based FRAs are significantly affected by the users’ perceptions of their complexity. According to Chuah et al. (2021), complexity is defined as the situation in which an innovation is perceived to be difficult to understand and use. Complexity can be further divided into (a) complexity of innovative idea (understandability) and (b) complexity of executing idea (usage) (Ram and Sheth, 1989). Some individuals are still fearful or scared to use any new disruptive technology and imagine as something frightening just like a monster (Chuah et al., 2021). A study by Belanche et al. (2019) has suggested that individual’s behavior toward the new robo-based technologies is quite complex and needs consideration both the designing and the traits of the individuals. This complexity can further lead to user resistance to robo-advisory services. In context of FRAs, investors may feel significant degrees of complexities in terms of understanding and effectively utilizing them. This might increase their strain and lead to resistance, resulting in higher stress levels. Studies have shown that whenever an individual’s cognitive load increases, it might lead to negative emotions and reluctance to engage with AI-based FRAs. However, an individual attitude toward AI might influence the extent to which the perceived complexity drives resistance toward FRAs. When discussing AI-based technology, Oprea et al. (2024) support the notion that a user with a more positive attitude toward AI would consider its intricacies inconsequential and vice versa. Thus, the moderating effort of AI between the perceived complexity and the resistance toward FRA also needs to be evaluated. Therefore, it proposes the following hypotheses:

*H1: Perceived complexity is positively related to resistance to FRA.*

*H1a: Perceived complexity and resistance to FRA is moderated by attitude towards AI.*

### 3.2 Value barrier and resistance to FRA

The term ‘value barrier’ denotes the reluctance toward an innovation due to lack of alignment with the benefits and existing value, specifically in balancing costs and perceived benefits (Lyu et al., 2024). To make FRAs appealing, the potential investors must recognize its value (Bhatia et al., 2021). In other words, without apparent value, the resistance toward innovative services would be a natural response (Ram and Sheth, 1989). When the perceived costs exceed the perceived benefits, then the value barrier arises. In context of FRAs, potential investors may resist the use of these platforms, if they doubt the algorithm transparency, or other personalized insights which are previously offered by the traditional human advisors. Increased perceived costs over its perceived benefits is one of the major reasons that hinders the adoption of an innovative service as stated in a study by Kaur et al. (2020). Although FRAs provide numerous benefits, they are unable to answer the queries of the potential users about platforms ability to deliver worthy financial outcomes raising concerns and psychological resistance by the investors. However, the degree of the resistance may not be same/uniform among all investors as their attitude toward AI could play a crucial role in influencing this relationship. The investors with a positive attitude and mindset toward AI may be more willing to re-consider the value provided by FRAs, thus reducing the impact of value barriers on resistance. On the contrary, those who are skeptical or afraid of AI may suffer a heighten resistance, even if the value propositions are strengthen. Thus, building on the following, the study proposes the following hypotheses:

*H2: Value barrier is positively related to resistance to use FRA.*

*H2a: Value barrier and resistance to FRA is moderated by attitude towards AI.*

### 3.3 Data privacy risk and resistance to FRA

Data privacy risk is an individual’s anxiety about the potential threats about their personal information while utilizing a certain system or service. This anxiety stems from the fear that any unauthorized use of sensitive data may lead to harm or misuse. Data privacy risks might lead to negative emotions on the part of the individual doubting the ability of the technology-based services. Numerous studies have done research on privacy risk factors such as facial payment recognition systems (Liu et al., 2021), home IOT systems (Lee, 2020), and smart services (Mani and Chouk, 2022). In context to information system studies, data privacy risks are one the most vital factor that acts as a barrier in accepting automated-based technologies such as FRAs. Thus, the IRT model considers data privacy risk as a very vital barrier to use any innovative technology. In context of FRAs, they gather sensitive financial information, which makes them vulnerable to data breaches. The susceptibility of these digital platforms to data breaches may elevate investor concerns about the safety of their personal information leading to cognitive stress.

However, the investors with a difference in attitude toward AI can build up these perceptions significantly. Sindermann et al. (2021) suggest that individuals holding a strong and favorable attitude in AI reliability and efficiency can actually buffer the negative impacts of the perceived privacy concerns and vice-versa. Thereby, attitude toward the AI might play a moderating role in softening their resistance to use FRAs. Building on this, the study proposes a hypotheses:

*H3: Data privacy risks is positively related to resistance to use FRA.*

*H3a: Data privacy risks and resistance to FRA is moderated by attitude towards AI.*

### 3.4 Overconfidence bias and resistance to FRA

Overconfidence bias refers to a situation where individuals tend to overrate their knowledge, control over financial results, or predictive abilities, leading to cognitive distortion (Zheng et al., 2025). In other words, it is the difference between the subjective beliefs of an individual and objectively measurable outcomes (Piehlmaier, 2022). Potential investors who are overconfident prefer to depend on their own judgment, underrating the potential value of professional or algorithm-based management. The outlook to perceive that algo-based FRAs is inferior or irrelevant when compared with investors own knowledge and judgement generates psychological resistance to delegate sensitive financial decisions to automated devices, that to with minimal human interference. This further might leads to distrust in algorithm-based recommendations, when compared to their own set of intuitions. Previous studies also suggest that confidence actually influences the adoption and spread of innovation and vice-versa (Karki et al., 2024; Germann and Merkle, 2023; Piehlmaier, 2022). However, it is also a matter of consideration that not all of the investors perceive AI uniformly. Those investors with positive and more favorable attitude and confidence toward AI-based technologies may become more open to delegate their tasks to intelligent automated systems, even though if initially they had exhibited overconfidence. A study by Pal et al. (2025) also suggests that a favorable orientation toward using AI can help in reducing the algorithm aversion and vice-versa. This indicates AAI may moderate the relationship strength between overconfidence bias and resistance to FRAs. Therefore, building on this prospect, the following hypotheses are formed:

*H4: Overconfidence is positively related to resistance to FRA.*

*H4a: Overconfidence bias and resistance to FRA is moderated by attitude towards AI.*

### 3.5 Image barrier and *resistance to FRA*

Image barrier refers to the negative impression of the innovation, emerging when users perceive complications related to the use of the technology (Lee and Kim, 2022). In context of FRAs, perceived barriers emerge when investors view these AI-based technologies as overly opaque, unreliable, or complex due to their algorithmic foundations. This characterization of FRAs as black box lacking transparency in decision-making process leads to a situation of unpredictability. This perception might further provoke cognitive discomfort, confusion, or anxiety to use a new innovation. Previous literature also supports this image barrier results in users’ resistance to use mobile banking (Laukkanen and Kiviniemi, 2010), service robots (Lee and Kim, 2022), autonomous delivery vehicle (Lyu et al., 2024), and IOT (Mani and Chouk, 2018). Perceived image barriers in robo-advisory services emerge when users view these automated platforms as overly complex, opaque, or unreliable due to their algorithmic foundations. The characterization of FRAs as “black boxes” lacking transparency in their decision-making processes contributes to a perception of unpredictability and risk. This might lead to perceiving the AI-driven financial tools as inconsistent, impersonal in comparison to the traditional methods of financial advice. Notably, these image-related doubts about AI often build up specifically when individual beliefs undermine the utility of AI-driven financial services and do not align with their self-concepts and values, which may pose as a psychological reluctance toward these services. Yet, if an individual having a positive disposition toward AI might override the symbolic incongruities linked to FRAs. The more the strong positive attitude and belief toward the AI technology, the more will be the probability to offset the image-related barriers and enhance the openness to use technological alternatives such as FRAs. Thus, attitude of an individuals could play an important role in influencing the degree to which the image barriers can be translated toward resistance to use FRAs. Building on the above discussions, the following hypotheses are formed:

*H5: Image Barrier is positively related to resistance to FRA.*

*H5a: Image barrier and resistance to FRA is moderated by attitude towards AI.*

### 3.6 Inertia and resistance to FRA

Inertia refers to the tendency to stick with the existing system despite of using other better alternatives and showing resistance to change (Zhang et al., 2024). This inertia can impact the individuals, enhancing beyond functional barriers to address psychological obstacles to innovation. The psychological inertia typically has a tendency to maintain status quo (Samadi et al., 2024). In other words, inertia stems from the unique challenge to reframe the existing ideas and established traditions on the basis of innovative ideas (Schmid, 2019). In context of FRAs, inertia can actually reduce the tendency to believe in the algorithmic intelligent systems leading to psychological changes and favor their existing systems (Danneels et al., 2018). In other words, the investors might stick to their traditional advisory and self-directed methods which might create performance doubts about the FRAs. Previous studies have also validated this, such as healthcare professional resistance (Zhang et al., 2024), AI chatbots (Xi, 2024), and socio-technical inertia (Schmid, 2019). Still, research suggests that this impact might not be same for all the individual investors. There is a possibility that the investors with a positive outlook toward AI are more open to re-establish the routines and may perceive AI-driven systems as an empowering tool rather than disruption (Pal et al., 2025). Their belief system in AI-based technologies such as FRAs may help them counter their inertia that is rooted in traditional practices. Thus, attitude toward the AI might moderate the relationship between inertia and resistance to use FRAs either by reinforcing or dampening their intentions to use FRAs. Building on the same, the following hypotheses can be formed:

*H6: Inertia is positively related to resistance to FRA*

*H6a: Inertia and resistance to FRA is moderated by attitude towards AI*

### 3.7 Resistance to FRA and intention to use and recommendation intention

Resistance denotes the psychological state of reluctance or aversion caused by conflicting ideas or beliefs when confronted with novel systems, which has a significant influence on user behavior in technology adoption contexts (Mani and Chouk, 2022). This reluctance may stem from skepticism about the financial decision-making by the algorithms or may be due to discomfort with reduced human interactions or may be due to perceived threats. This dissonance might cause negative emotions such as anxiety or confusion, which can further impact their behavioral intentions. Such dissonance not only leads to resistance but also reduces an investor’s intention to accept, further lowering down their willingness to even recommend these services to other users. Previous studies have shown resistance negatively impacts on the user willingness to continue mobile apps (Migliore et al., 2022). Similarly, a study on technology renewal revealed that reasons for user resistance and new information technology (Shirish and Batuekueno, 2021), resistance to change in healthcare (Shahbaz et al., 2019), and another study on O2O platforms resistance by the small retailers (Chawla et al., 2024a; Chawla et al., 2024b; Jafri et al., 2025) have positive impact on discontinuous intentions. Prior attitudes toward AI-backed technologies are a significant predictor of user behavior, influencing how individuals process new information and whether they accept or reject it (Li, 2023). When users have positive prior attitudes towards AI-backed technologies, they are more likely to regard these platforms as efficient and trustworthy, thereby motivating for greater engagement. On the other hand, users with unfavorable or less prior attitudes may feel reluctant due to their conflicting beliefs about the transparency of FRAs. This emotional stress can lead to lowering their willingness to accept these AI-backed financial services. In context of FRAs, users might face cognitive dissonance when they feel uncertainty about the robo-advisors’ platforms to deliver relevant and reliable information conflicting with the desire to stick to their traditional practices. Building on this, we propose the following hypotheses:

*H7: Resistance towards FRA is negatively related to intention to use it.*

*H8: Resistance towards FRA is negatively related to recommendation intention.*

In the world of finance and technology services, where investors frequently feel anxious and uncertain about their finances, robo advisory services which are entirely based on algorithms driven advice system may provoke resistance.

**Figure 1:**
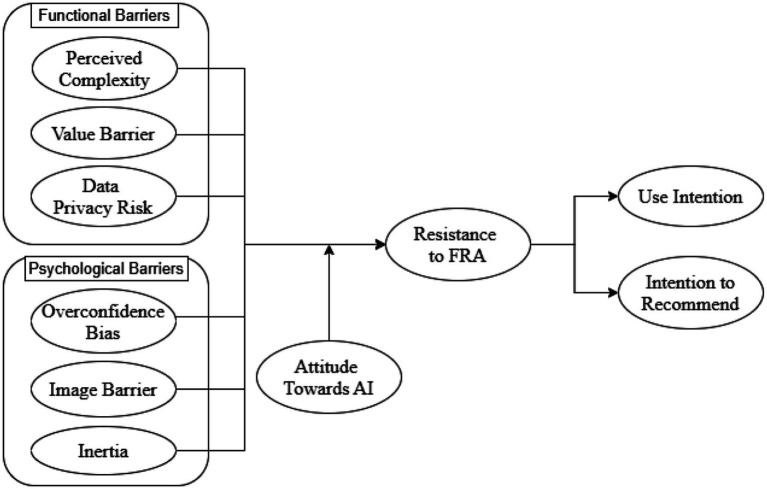
Conceptual model.

Form the above laid literature, the current studies formulate this conceptual model (see Figure 1).

## 4 Methodology

### 4.1 Instrumentation

To test the conceptual framework, the study uses India as a geographical setting. Measurement scales from previously published research were used to operationalize the scale items of the identified components. Using the earlier research of Parissi et al. (2019), items of perceived complexity were assessed. The scale items for inertia and perceived security risk were assessed from Mani and Chouk (2018). The standardized measures for over-confidence were taken from Meyer et al. (2013). Items from the research by Chawla et al. (2024a) and Chawla et al. (2024b) were used to get the scale items for the construct value barrier and image barrier. The resistance to use scale items was obtained from research conducted by Chawla et al. (2024a) and Mani and Chouk (2018). The Sindermann et al. (2021) and Cheng et al. (2019) study served as the source of the standardized measures for attitude toward AI. Prior research by Chawla et al. (2024a) was used to evaluate the items of use intention. The items for recommendation intention were extracted from the study of Rahi et al. (2018). A five-point Likert scale was employed to measure each statement, with 1 denoting “strongly disagree” and 5 denoting “strongly agree.”

### 4.2 Preliminary testing and data collection

To assess the items chosen for the investigation, a screening test was conducted with an expert panel consisting of three professors from a reputed state university, as subject-matter experts and four Fintech industry experts from North Indian states. To arrive at the final instrument, two to three discussion sessions were held both in person and electronically via Google Meet. Industry experts recommended the inclusion of individual’s attitude toward AI. In the second step, pilot testing was undertaken, with the questionnaire distributed to a total of 70 research scholars and academics via Google Form as well as in person. This cohort was asked to score the scale items and recommend any things that may be added or alter to increase clarity. A total of 54 respondents filled out the form and offered a few changes to the phrasing of scale items. There were no new scale elements introduced, but four were eliminated due to low mean values. The group’s comments were integrated into the questionnaire to increase its clarity.

Data collection was conducted both online and offline. Non-probability sampling approaches were used due to the lack of a sufficient sampling frame (Vehovar et al., 2016). Data were gathered using purposive and snowball sampling methods. The study opted for the non-probability purposive sampling as the study focused on the investors that were familiar with financial robo-advisors. Still, this method does not provide the full generalization, but the study considered to include the diverse group of respondents and applied very clear screening criteria. In addition, the study also acknowledged the possible biases and carefully interpreted the results, following the widely accepted guidelines (Hair et al., 2010). Purposive sampling is preferable in studies when respondents have little familiarity with the event under investigation (Sibona and Walczak, 2012). Because obtaining a list of all persons using or considering utilizing a financial robo-advisor was tough, data were obtained using the purposive and snowball sampling techniques proposed by Naderifar et al. (2017). The questionnaire was administered via platform such as LinkedIn, WhatsApp, and Telegram, particularly in forums and groups related to financial investment. Nonetheless, we sought individuals through personal and professional networks who had used or preferred to use FRA. Participants were also invited to share the survey link with others with similar profiles or recommend a few colleagues, relatives, and acquaintances, allowing for snowball sampling. Several reminders through emails and revisits were undertaken to approach the respondents. The sample size was chosen using Siddiqui et al. (2013) study, which concluded that 384 respondents are sufficient for up to 90 scale items. Accordingly, approximately 500 plus questionnaires were sent, and after confiscating unfinished responses, a final sample of 409 responses was obtained, with the respondents’ profiles presented in Table 1. This sample size exceeds the allowed limit of 384 and supports the robustness of the statistical analyses and findings. To exclude social desirability biases, the respondents were assured secrecy for their responses. They were also assured that their replies would only be utilized for scholarly reasons. The study’s goals were explained to participants at the outset, and a detailed explanation of robo-advisors was provided to encourage true and candid responses. In addition, respondents were given assurances of data security, anonymity, and confidentiality to protect their privacy and foster confidence. Table 1 depicts the demographic characteristics of the research participants. The bulk of responses are male (71.51%). In terms of age distribution, approximately 60% are between the ages of 25 and 44. Approximately half of respondents (48.17%) had at least a bachelor’s degree, with 34.47% holding a master’s degree. The profile also includes statistics for approximately 48% of respondents with a bachelor’s degree, followed by 34.47 as master degree holders. Furthermore, the majority (33.25%) claimed 1–3 years of investing experience, followed by 27.38% with 4–6 years of experience.

**Table 1:** Respondents’ demographic profile.

Variable (*N* = 409)	Characteristics	Count	Percentage
Gender	Male	291	71.15
	Female	118	28.85
Age (in years)	18–25	103	25.18
	25–35	141	34.47
	35–45	101	24.69
	45–55	54	13.20
	Above 55	10	2.44
Education qualification	Bachelor’s degree	197	48.17
	Master’s degree	141	34.47
	Professional degree (CA, CFA, etc.)	30	7.33
	Doctorate	41	10.02
Profession	Student	87	21.27
	Salaried employee (private)	154	37.65
	Salaried employee (Government)	59	14.43
	Self-employed/business	103	25.18
	Retired	6	1.47
Investment experience (in term of no. of years)	Less than 1 year	63	15.40
	1 to less than 4 years	136	33.25
	4 to less than 7 years	112	27.38
	7 years and above	98	23.96

Source: Authors’ compilation.

## 5 Data analysis and results

### 5.1 Initial quality checks of data

Various prerequisite quality checks were performed before simply moving on to final data analysis. These tests were followed by the measurement model and structural equation modeling was performed using SPSS and AMOS. As recommended by Byrne (2013), the arithmetic mean was used to replace the missing data. Data were checked for non-response bias and to do that the mean differences between the initial 50 responses and the final 50 responses out of all were assessed. Non-response bias was not an issue as no statistical difference was found. The data were further tested to see whether common method bias (CMB) exists or not. Harman’s single-factor test was utilized to test this (Harman, 1976; Shkoler and Tziner, 2017). Assuming that the occurrence of either a general factor or a single factor accounting for the majority of covariance among measures indicates the existence of CMB. This entails: combining all scale items into a single factor with varimax rotation in exploratory factor analysis (Podsakoff et al., 2003, p. 889). It is recommended that the single-factor solution’s explained variance must not exceed 50% (Harman, 1976). The outcomes shown in Appendix Table A.1 display a single factor variance value of 28.570%, which is less than the suggested value. This indicates the absence of CMB.

To assess the collinearity among constructs, the study additionally evaluated for multicollinearity in accordance with the recommendations proposed by Hair et al. (2021). The variance inflation factor (VIF) values were computed to do this. The range of VIF values required to exhibit multicollinearity is 0.20 to 5.0. All of the VIF values, as shown in Appendix Table A.2, fall between 1.29 and 2.38, which is the optimal range and indicates that multi-collinearity is not a problem.

### 5.2 Reliability and validity of the instrument

Confirmatory factor analysis (CFA), which indicates hypothesized causal connections between latent and observed indicator variables, was used to make sure fit among observed data and a theoretically grounded model to ensure reliability and validity criteria prior to the structural model assessment (Hancock and Mueller, 2001, p. 5240). The factor loadings of the constructs and average variance extracted (AVE) were taken into consideration to assess the convergent validity of the exogenous and endogenous constructs (Hair et al., 2010). Items with standardized factor loadings of 0.6 or above are considered appropriate (Kline, 2005). Adequate convergent validity is indicated by AVE values more than 0.5 (Bagozzi et al., 1991; Fornell and Larcker, 1981). Composite reliability was calculated to address internal consistency, i.e., reliability. According to Fornell and Larcker (1981), the value of 0.7 for composite reliability is appropriate. In addition, as recommended by Hair et al. (2010), correlation was carried out to verify discriminant validity. According to Hair et al. (2010), the suggested values of AVE should be greater than inter-item correlations, indicating that constructs are not heavily associated. Following the determination of a suitable factor structure, structural equation modeling was used to evaluate the hypothesized correlations between exogenous and endogenous constructs in the study.

From Table 2, it is evident that item loadings for all constructs range from 0.703 to 0.939, which are over the recommended threshold value, i.e., 0.60 (Kline, 2005). All of the scale items’ critical ratio values are greater than 1.96, suggesting that the data are normally distributed (Byrne, 2013). Convergent validity is thus demonstrated by these findings. In addition, Table 2 reports on the validity and reliability of the constructs and the scale items associated with them. The composite reliabilities of the constructs, which fall between 0.842 and 0.951, all exceed the threshold of 0.7, illustrating internal consistency. The fact that each construct’s AVE is higher than 0.5 confirmed convergent validity and further supports overall model validity (Fornell and Larcker, 1981).

**Table 2:** Measurement model.

Construct	Items	Estimate	S.E.	C.R.	CR	AVE	α
Perceived complexity	PPC1	0.714	0.055	13.283			
	PPC2	0.939	0.04	29.782	0.932	0.738	0.928
	PPC3	0.945	0.041	27.768			
	PPC4	0.874	0.044	23.703			
	PPC5	0.85					
Value barrier	VAB1	0.871	0.052	16.56	0.892	0.7044	0.888
	VAB2	0.89	0.056	17.23			
	VAB3	0.75					
Data privacy risk	DPR1	0.826	0.08	14.478			
	DPR2	0.864	0.084	14.669	0.842	0.642	0.838
	DPR3	0.705					
Image barrier	IMB1	0.94	0.027	36.756	0.951	0.866	0.944
	IMB2	0.906	0.029	32.719			
	IMB3	0.946					
Overconfidence bias	OCB1	0.828	0.069	15.806			
	OCB2	0.85	0.067	15.941	0.855	0.664	0.854
	OCB3	0.764					
Inertia	INE1	0.891					
	INE2	0.779	0.051	17.534	0.845	0.581	0.842
	INE3	0.748	0.051	16.682			
	INE4	0.703	0.053	12.677			
Resistance toward FRA	RRA1	0.858					
	RRA2	0.85	0.048	20.769	0.896	0.741	0.891
	RRA3	0.874	0.047	21.5			
Attitude toward AI	AAI1	0.882					
	AAI2	0.788	0.042	18.241			
	AAI3	0.757	0.042	16.972	0.865	0.620	0.862
	AAI4	0.712	0.044	13.167			
Use intention	INU1	0.879					
	INU2	0.871	0.048	19.17	0.854	0.777	0.852
	INU3	0.895	0.047	20.5			
Intention to recommend	INR1	0.861					
	INR2	0.905	0.042	22.451	0.819	0.76	0.815
	INR3	0.849	0.043	21.145			

Model fit indices (CMIN/df = 2.441, GFI = 0.94, NFI = 0.911, CFI = 0.928, TLI = 0.927, IFI = 0.935 and RMSEA = 0.071).

To ensure discriminant validity, correlation analysis of the constructs was performed. To ensure the discriminant validity, the square root of AVE must be more than inter-item correlations, which means the constructs are not highly correlated (Hair et al., 2010). Table 3 demonstrates that the square root of all AVE values is higher than the inter-item correlations and hence indicates that the measurement model has sufficient validity and the model is appropriate for further structural testing.

**Table 3:** Discriminant validity.

Construct	Image barrier	Perceived complexity	Value barrier	Overconfidence bias	Data privacy risk	Resistance to FRA	Inertia	Attitude toward AI	Use intention	Intention to recommend
Image barrier	**0.931**									
Perceived complexity	0.212	**0.859**								
Value barrier	0.213	0.125	**0.826**							
Overconfidence bias	0.209	0.157	0.243	**0.815**						
Data privacy risk	0.418	0.31	0.168	0.094	**0.801**					
Resistance to FRA	0.394	0.467	0.321	0.182	0.334	**0.861**				
Inertia	0.41	0.198	0.148	0.177	0.363	0.314	**0.762**			
Attitude toward AI	0.454	0.407	0.381	0.122	0.394	0.301	0.206	**0.787**		
Use intention	0.321	0.289	0.178	0.351	0.267	0.27	0.312	0.305	**0.793**	
Intention to recommend	0.235	0.112	0.198	0.214	0.156	0.218	0.189	0.278	0.356	**0.821**

Source: Authors’ calculations. The bold value represents the square root of AVE, and the off-diagonal values represent inter-construct correlations for respective variables.

## 6 Findings and discussion

**Figure 2:**
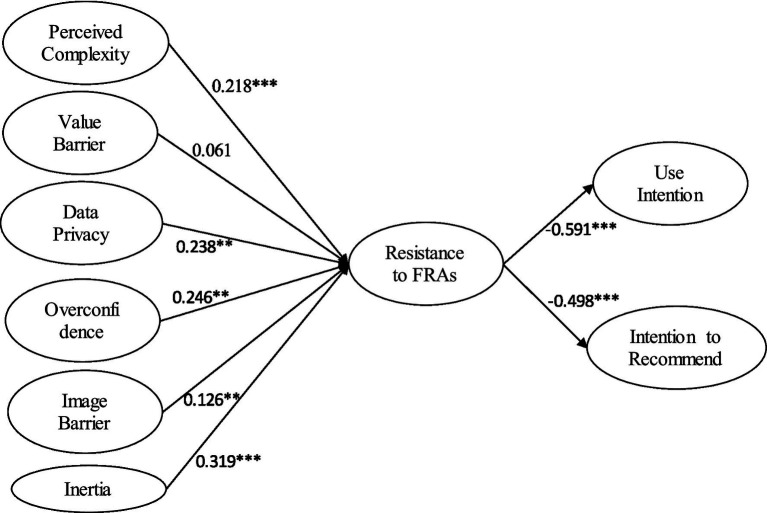
Path analysis.

Figure 2 and Table 4 provide the results of structural model. Model fit indices indicate an adequate model fit (CMIN/df = 3.147, GFI = 0.931, NFI = 0.902, CFI = 0.930, TLI = 0.921, IFI = 0.931, and RMSEA = 0.073). The findings provide strong statistical support for the theoretical premise that investors’ reluctance to use FRA is greatly influenced by functional and psychological barriers, which in turn further impacts their intention to use and recommend FRA.

**Table 4:** Path analysis.

Hypotheses	Independent variable	Dependent variable	Std. β	t-Stat.	Decision
H1	Perceived complexity	Resistance to FRA	0.218***	7.968	Supported
H2	Value barrier		0.061	1.203	Un-supported
H3	Data privacy risk		0.238**	2.35	Supported
H4	Overconfidence bias		0.246**	2.607	Supported
H5	Image barrier		0.126**	1.983	Supported
H6	Inertia		0.319***	9.767	Supported
H7	Resistance to FRA	Use Intention	−0.591***	19.45	Supported
H8	Resistance to FRA	Intention to Recommend	−0.498***	16.56	Supported

Source: Authors’ calculations.

Among the functional barriers, perceived complexity (H1) associated with the service emerged as a strong influencer of resistance to FRA. The findings revealed that when individuals perceive the platform interface and the processes involved as difficult to comprehend and navigate with, their resistance to FRA intensifies. This finding goes well with the studies that emphasize the importance of transparency and usability associated with the technology product (Gomber et al., 2018; Cheng and Mitomo, 2017). The black box nature and the potential conflict of interest associated with the algorithm decision-making make individual reluctant as they are unknown of methodology and rationale behind portfolio recommendations (Saivasan, 2024; Aw et al., 2023). Nevertheless, complexity while interacting with the platform can intensify perceived effort, thus, reducing the cognitive convenience desired form such platforms (Mirhoseini et al., 2024).

Contrary to that, the study found an insignificant linkage of value barrier with resistance (H2). The outcome, however, diverges from the existing traditional technology-based literature, which advocate that lack of perceived value commonly impacts resistance (Chawla et al., 2024a). But when it comes to FRAs, the results make sense when you take into account how inexpensive, convenient, and easily accessible these platforms are. In contrast to traditional human advisors, this AI-backed FRAs charges considerably lower fees and offer sophisticated algorithm-based personalized advice for small retail investor (Onabowale, 2024). It may be argued that the person understands the benefits of FRA; hence, their resistance to this technology may not be due to a value barrier.

Data privacy risk (H3) came out as a strongest functional barrier to resistance, indicating apprehension about how AI-based investing platforms manage customers’ private financial information. This aligned well with the broad spectrum of the studies that highlights privacy concerns as a critical factor for every data-lead innovation (Javadi, 2024). Especially the financial domain magnifies these concerns as the misuse of user’s data may result in potential reputational and monetary losses. The recent recurrent topics being examined in the literature, such as third-party data sharing and data governance transparency, which might account for user reluctances (Lie et al., 2022; Wiseman et al., 2019), lend credence to this finding.

Discussing the psychological barriers, the hypothesis H4, i.e., the over confidence barriers, significantly influences resistance toward FRA. Individuals who overvalue their own financial intelligence as well as their market prediction skills do not honor or accept the algorithmic advices, assuming that they can take better financial decision by their own. This finding converges well with the prior studies arguing that overconfidence as a cognitive distortion, restrict the individual to rely on external available tools and support systems (Jermias, 2006). One’s belief in their superior judgement diminishes adoption of automation, even though these systems turn out to be reliable, intelligent, and impartial.

Image barrier (H5), another psychological barrier, had a modest yet significant impact on resistance. The possible explanation for this outcome is the image associated with FRAs, as they have been perceived as impersonal and suitable for either technology sophisticated or less wealthy retail investors, thus leading to symbolic reluctance toward FRAs. Given the current geographical context of the study, i.e., India and its socio-cultural settings where financial decisions are knotted with relationships and trust, such perception certainly leads to adoption resistance (Cardillo and Chiappini, 2024). Nonetheless, the comparatively decreased strength of this link would suggest that FRA technological platforms may becoming more accepted, particularly among younger and urban groups.

The hypothesis H6 inertia emerged as the strongest barrier influencing resistance, which is in line with status quo bias literature (Koh and Yuen, 2025; Martin, 2017). It advocates that irrespective of possible gains, individuals often do not alter their status quo due to their comfort with the traditional advisory practices. The literature has extensively documented inertia against new innovations, especially in the Fintech sector, where even pleased and existing users of digital systems exhibit reluctance to the introduction of new forms or models (Polites and Karahanna, 2012). When it comes to financial decision-making in particular, habits developed over time can make the transition to a new system emotionally and intellectually burdensome.

The linkage between resistance and intent to use FRA was negative and statistically significant, supporting the common belief that resistance acts as a direct barrier to behavioral adoption. This is in line with the existing behavioral models/frameworks where resistance is shown as a pioneering behavioral reluctance (Chawla et al., 2024a; Kaur et al., 2020). Similarly, the intention to recommend FRA was negatively influenced by resistance, indicating that the user not only abstains from adopting FRAs for their usage but also not recommends it to others. This finding offers insights as this effect is especially important given digital services where the influence of peers and referrals critical role in technology adoption (Kaur et al., 2020). These results highlight the urgent need to overcome resistance as a crucial strategic obstacle to encouraging the use of FRAs.

### 6.1 Testing moderation

To test the hypotheses, moderation test was performed. Table 5 indicates the effects of moderation where attitude toward AI does not moderate the relationship of perceived complexity, value barrier, and image barrier with resistance. This indicates that user’s having favorable attitude toward AI is not immune to challenges such as complex interfaces or stereotype associated with FRAs. Significant moderating effects of attitude toward AI were seen in the cases of inertia, overconfidence bias, and data privacy risk. This implies that users’ resistance is less affected by the data privacy risk if they have supportive disposition to AI. Because of this, users’ perceived vulnerability may be lessened (Oprea et al., 2024). In addition, in case of overconfidence bias, user’s reluctance toward FRAs is reduced as their favorable attitude toward AI will strengthen their belief in the superiority of AI-backed decision-making (Heidari, 2024). Finally, the relationship between inertia and resistance is also moderated by attitude toward AI, inferring that positive AI beliefs could alter habitual resistance and drive openness to change (Balakrishnan et al., 2024).

**Table 5:** Attitude toward AI as a moderator.

Hypotheses		High	Low	Diff.	Result
H1a	Perceived Complexity → AAI(Moderator) → Resistance	0.256	0.196	0.78	Not-supported
H2a	Value Barrier → AAI(Moderator) → Resistance	0.368	0.222	1.9	Not-supported
H3a	Data Privacy Risk → AAI(Moderator) → Resistance	0.249	0.112	1.99	Supported
H4a	Overconfidence Bias → AAI(Moderator) → Resistance	0.605	0.422	2.16	Supported
H5a	Image Barrier → AAI(Moderator) → Resistance	0.5	0.375	1.75	Not-supported
H6a	Inertia → AAI(Moderator) → Resistance	0.578	0.454	1.98	Supported

Source: Authors’ calculations.

## 7 Conclusion and implications

### 7.1 Conclusion

On the psychological basis, user resistance was prominently influenced by overconfidence bias, image barrier, and inertia. These findings revealed that cognitive bias, identity associations, and a dependence on conventional financial practices continue to impede widespread adoption of robo-advisors’ platforms. Essentially, resistance itself had a negative and significant influence on both the usage intentions and recommendation to FRAs, validating resistance as a key barrier in user decision-making process. The study presents a nuanced and empirically validated knowledge of the key barriers driving reluctance to financial robo-advisor adoption in Indian context. The findings, drawn from a sample of 409 respondents and analyzed using structural equation modeling (SEM), confirm that both functional and psychological blockades influence resistance to robo-advisory services. Among the functional barriers, data privacy risk and perceived complexity were shown to considerably increase user reluctance, underscoring ongoing concerns about usability and the management of sensitive financial data. Conversely, value barrier which is often seen as a central factor of innovation resistance was found to be minimal. This finding demonstrates rising consumer awareness and knowledge of the low-cost, accessibility, and other concrete benefits of robo-advisory platforms, specifically when contrasted to conventional human financial advisors.

In addition, to enhance the model, the study tested the moderating influence of attitude toward AI on the relation between individual barriers and resistance. Noteworthy, this moderating impact was only observed in cases of data privacy risk, overconfidence bias, and inertia, implying that those individuals with a more positive attitude toward AI are less resistant, even in the face of these specific problems. Nevertheless, no significant moderation was discovered for value barrier, perceived complexity, and image barrier, suggesting that technological optimism alone may not be enough to overcome perceived usability difficulties or social-symbolic reluctance.

### 7.2 Implications

#### 7.2.1 Theoretical implications

The study makes several vital theoretical contributions to the literature on Fintech adoption, specifically in relation to robo-advisory platforms. The study integrates innovation resistance theory (IRT) with moderating lens of attitude toward AI, advancing our knowledge on how both the barriers—functional and psychological impacts user reluctance to technology—based financial services. Previous literature has frequently explored adoption through the lens of factors such as ease of use, trust, and perceived usefulness (e.g., Sironi, 2016; Belanche et al., 2019), but this study shifts its focus toward the hindrances, thereby providing a counter-perspective that augments comprehensive discourse on digital reluctance.

Furthermore, the empirical findings that the value barrier in this context is insignificant call into question and challenge the traditional assumption of perceived economic trade-off as a critical deterrent, implying that in high-tech, low-cost service domains such as FRAs, behavior inertia and psychological discomfort might overshadow rational cost–benefit analysis.

The addition of attitude toward AI as a moderator widens the explanatory power of IRT, by identifying that users’ cognitive and emotive orientation toward AI systems may increase or reduce perceived barriers to reluctance and consequent behavior intentions. As a result, the study majorly contributes an integrated framework that may be adopted or expanded in future research on technology resistance across different disciplines.

#### 7.2.2 Practical implications

The outcomes of the study have vital implications for the various stakeholders involved in the designing, promotion, regulation, and adoption of financial robo-advisors (FRAs), specifically within the Indian investment context. The study observes the significant impact of both barriers, i.e., functional and psychological barriers, along with the moderation of attitude toward the AI, and it becomes quite vital that the stakeholders adopt evidence-driven and nuanced strategies for the promotion of FRAs.

The present financial institutions must recognize that the resistance toward the FRAs is more impacted by the psychological factors rather than the functional factors. Particularly, inertia as a factor emerged as one of the most impactful predictors of the resistance toward the FRA, which is followed by the overconfidence biases. The results of the study further imply that the traditional methods of awareness programs might fell short until they are complemented by the behavioral interventions. In addition, the service providers should also implement the tactics such as gamification, nudging, and using default options to help the users in reducing their inertias and bringing ease to the users of robo-advisory platforms. The concerns related to the data privacy also warrant a special attention. To enhance the user, trust the transparent data practices, visible third-party security and user-controlled privacy settings can be helpful. Furthermore, this resistance significantly decreases the usage as well as the recommendation intentions; the firms should require to prioritize the attitude building initiatives such as educational outreach, hybrid advisory models, and testimonials for the early users of FRAs, thereby lowering the psychological discomfort and increasing the trust levels. In addition, the significant influence of data privacy risk on the resistance further indicates that there is an urgent requirement for robust AI-based financial data protection structures. The policymakers need to turn their focus on strengthening their cybersecurity norms and also implementing the transparency mandate for these AI-based financial technologies.

The moderating role of the AAI advocates that attitude of the individuals toward AI needs to be addressed systematically. Specifically, in a country like India, national level digital literacy and AI-awareness programs are required to be rolled out, particularly targeting the Tier-II and Tier-III cities where trust issues are more pronounced. Furthermore, the simplification of the redressal of grievance procedure for digital financial products can also act as a deterrent to misuse while boosting the confidence levels of the investors.

The developers must focus on system transparency and user experiences. The lack of moderation impact of the AAI on the perceived complexity, image concerns, and the value barriers highlights that these issues cannot be resolved merely through positive attitudes but by prioritizing the real-time feedbacks (explainable AI), transparency in performance metrics to make the users feel informed and controlled. Notably, this moderation effect of AAI on the psychological factors, i.e., inertia, overconfidence underscores the opportunity for technology to support rather than challenging the user’s autonomy to take decisions. For scenario analysis, users personalized advisory pathways, adjustable risk settings, and co-piloting interfaces can serve to resonate with AI outputs with preferences of the user curtailing distrust and defensiveness.

For Indian retail investors, the findings underscore the role of self-awareness of the individuals in financial decision-making. The resistance is not only specifically based on the technology-related flaws but it is significantly influenced by the behavioral inertia and personal biases. The investors are encouraged to participate in the workshops and engage in peer learning networks and self-reflection about their resistance toward adopting these digital financial advisory services. By working on their own attitudes toward the AI can help in the comprehension of its functional workings, Indian investor can make more balanced and informed choices, which are essential requirements in a rapidly digitizing financial landscape.

#### 7.2.3 Limitations

Despite providing valuable insights on the challenges to FRA adoption, the study has certain drawbacks. First, the research is limited to India, a developing market with unique economic, socio-cultural, and technological characteristics that may not be generalized to other countries. While India is growing rapidly as a digital nation, yet it is not unable to get out of its conventional and conservative financial ecosystem. Moreover, the user behavior and attitude in technologically mature nations might differ significantly. Second, the potential bias can be introduced due to the self-reported data, leading to inaccuracy, specifically when users judge their own resistance or cognitive bias such as “overconfidence.” Furthermore, the study design is based on cross-sectional methodology which simply restricts the causal inferences, and longitudinal approaches that might capture transitions in the perception and behavior overtime as users gain more experience and exposure of robo-advisory platforms. Finally, while the SEM—structural equation modeling—provides a rigorous way to explore correlation between the variables, latent constructs (e.g., resistance and attitude toward AI), it may display deeper psychological complexity that quantitative methods cannot provide completely.

#### 7.2.4 Future research scope

Building on the study findings and limitations, several promising avenues for future research directions emerge. First, to track the resistance and adoption behavior in context of FRAs, longitudinal studies can be undertaken. Such longitudinal studies can determine how the experiences amend initial resistance and whether psychological and functional barriers are reduced with time. Second, the future research can do comparative cross-sectional investigations that involves emerging and developing economies revealing that how cultural aspects such as collectivism, building trust in automation, or other uncertainties influence the weight of specific barriers and the role of AI attitudes. Third, the model can be expanded in the future by incorporating mediators, or alternative moderators, such as algorithmic transparency, digital financial literacy, or financial concerns, to better understand the reasons for the resistance. In addition, for deeper insights to understand the reasons for resistance, the future work can adopt the qualitative research design, through interviews or focus group discussions, especially in concern to the emotional responses to automated services and perceptions of AI in financial services. Finally, if FRAs grow to integrate generative AI, such as voice-based interfaces, or hybrid human-AI models, future research studies must focus on how such advances reshape resistance dynamics, perhaps giving rise to new enablers or barriers that are not covered in the current framework.

## Data Availability

The raw data supporting the conclusions of this article will be made available by the authors, without undue reservation.
