# Supplementary material for: Artificial intelligence attitudes and resistance to use robo-advisors: exploring investor reluctance toward cognitive financial systems
Source: Front Artif Intell. 2025 Sep 17;8:1623534. doi: 10.3389/frai.2025.1623534 (PMC12484032; doi:10.3389/frai.2025.1623534)
Supplement: Supplementary file 1 [file Data_Sheet_1.docx]

**Appendix Table A.1** Harman’s Common Method Variance Test

| Component | Initial Eigenvalues | | | Extraction Sums of Squared Loadings | | |
| --- | --- | --- | --- | --- | --- | --- |
|  | Total | % of Variance | Cumulative % | Total | % of Variance | Cumulative % |
| 1 | 8.00 | 28.57 | 28.57 | 8.00 | 28.57 | 28.57 |
| 2 | 3.40 | 12.13 | 40.70 |  |  |  |
| 3 | 3.10 | 11.08 | 51.78 |  |  |  |
| 4 | 2.05 | 7.31 | 59.09 |  |  |  |
| 5 | 1.84 | 6.56 | 65.65 |  |  |  |
| 6 | 1.45 | 5.19 | 70.84 |  |  |  |
| 7 | 1.41 | 5.02 | 75.86 |  |  |  |
| 8 | 1.23 | 4.39 | 80.25 |  |  |  |
| 9 | 0.72 | 2.56 | 82.81 |  |  |  |
| 10 | 0.58 | 2.06 | 84.87 |  |  |  |
| 11 | 0.52 | 1.85 | 86.72 |  |  |  |
| 12 | 0.48 | 1.72 | 88.44 |  |  |  |
| 13 | 0.40 | 1.43 | 89.87 |  |  |  |
| 14 | 0.38 | 1.35 | 90.22 |  |  |  |
| 15 | 0.35 | 1.20 | 91.63 |  |  |  |
| 16 | 0.33 | 1.17 | 92.39 |  |  |  |
| 17 | 0.28 | 1.01 | 93.40 |  |  |  |
| 18 | 0.27 | 0.97 | 94.37 |  |  |  |
| 19 | 0.26 | 0.93 | 95.30 |  |  |  |
| 20 | 0.23 | 0.80 | 96.10 |  |  |  |
| 21 | 0.21 | 0.74 | 96.84 |  |  |  |
| 22 | 0.19 | 0.66 | 97.50 |  |  |  |
| 23 | 0.14 | 0.52 | 98.02 |  |  |  |
| 24 | 0.13 | 0.45 | 98.47 |  |  |  |
| 25 | 0.12 | 0.44 | 98.62 |  |  |  |
| 26 | 0.12 | 0.42 | 98.89 |  |  |  |
| 27 | 0.09 | 0.33 | 99.22 |  |  |  |
| 28 | 0.09 | 0.32 | 99.54 |  |  |  |
| 29 | 0.08 | 0.29 | 99.83 |  |  |  |
| 30 | 0.05 | 0.17 | 100.00 |  |  |  |

Source: Authors’ calculation. Extraction Method: Principal Component Analysis.

**Appendix Table A.2** Variance inflation factor.

| Construct | VIF | VIF | VIF |
| --- | --- | --- | --- |
| Perceived Complexity (PPC) | 1.67 | 1.34 | 1.56 |
| Value Barrier (VAB) | 2.08 | 1.96 | 1.66 |
| Data Privacy Risk (DPR) | 2.14 | 1.92 | 2.38 |
| Image Barrier (IMB) | 1.75 | 1.68 | 2.24 |
| Overconfidence Bias (OCB) | 1.87 | 1.91 | 1.34 |
| Inertia (INE) | 1.80 | 1.29 | 1.85 |
| Attitude towards AI (AAI) | 2.05 | 1.80 | 2.06 |
| Resistance Towards FRA (RRA) | | 1.69 | 1.85 |
| Dependent Variable => | Resistance Towards FRA (RRA) | Use Intention (INU) | Intention to Recommend (INR) |

Source: Authors’ Calculations

**Appendix Table A.3:** Instruments and Sources

| **Construct** | **Statements** | **References** |
| --- | --- | --- |
| Perceived Complexity | PPC1: Learning to use financial robo-advisors is easy for me. (R) | Parissi et al., (2019) |
|  | PPC2: I find financial robo-advisors to be flexible to interact with. (R) |  |
|  | PPC3: I find it easy to get financial robo-advisors to do what I want to do. (R ) |  |
|  | PPC4: It is easy for me to become skilful at using financial robo-advisors. (R) |  |
|  | PPC5: My interaction with financial robo-advisors is clear and understandable. (R) |  |
| Value Barrier | VAB1: FRA technology platforms would probably increase the cost of financial operations | Chawla et al., (2024) |
|  | VAB2: The cost of adopting FRA platforms outweighs the advantages they offer. |  |
|  | VAB3: FRA technology platforms would probably not satisfactorily deal with my financial problems |  |
| Data Privacy Risk | DPR1: The risk of an unauthorized third party overseeing the payment process is high | Chouk and Mani, (2019) |
|  | DPR2: The risk of abuse of usage information (e.g. credit card number, bank account data) is high when using FRAs |  |
|  | DPR3: The risk of abuse of billing information (e.g. credit card number, bank account data) is high when using FRAs |  |
| Overconfidence Bias | OCB1: How would you rate the likelihood of making a financial mistake if you were to manage this investment without the robo-advisor? | Meyer et al., (2013) |
|  | OCB2: How much would you rely on the presented robo-advisor in making this investment decision? |  |
|  | OCB3: How would you rate the amount of financial risk factors involved in this investment scenario? |  |
| Image Barrier | IMB1: I have a very negative image of FRA technology platform service | Chawla et al., (2024) |
|  | IMB2: FRA technology platforms would probably often be too complicated to be useful |  |
|  | IMB3: I have an image that FRA technology platforms are difficult to use |  |
| Inertia | INE1: I generally consider the change as a negative thing | Mani and Chouk, (2018) |
|  | INE2: I’d rather do the same thing rather trying the new ones |  |
|  | INE3: In my opinion past technological products were satisfactory so far |  |
|  | INE4: Overall, I consider that my needs in technological field have been met by existing technological products |  |
| Attitude towards AI | AAI 1: I think the application of AI technology will improve our quality of life. | Sindermann et al., (2021); Cheng et al., (2019) |
|  | AAI2: I believe AI will make my routine task easier. |  |
|  | AAI3: I would like to try to use AI-enabled product and services. |  |
|  | AAI4: I think intelligent products are relatively mature and rarely make serious mistakes |  |
| Resistance to Use FRA | RRA1: I have a negative opinion about the FRAs | Mani and Chouk, (2019) |
|  | RRA2: I’m not in favor of the FRAs |  |
|  | RRA3: I have a bad judgment on the FRAs |  |
| Use Intention | INU1: I intent to use Financial Robo-Advisors technology platform for my financial investments | Chawla et al., (2024) |
|  | INU2: I am likely to use Financial Robo-Advisors technology platform for my financial investments |  |
|  | INU3: I plan to increasingly use Financial Robo-Advisors technology platform for my financial investments |  |
| Intention to Recommend | INR1: I will recommend to my friends to use Financial Robo-Advisors technology platform | Rahi et al., (2018) |
|  | INR2: If I have a good experience with internet banking, I will recommend friends Financial Robo-Advisors technology platform |  |
|  | INR3 I will definitely recommend to my friends to use Financial Robo-Advisors technology platform |  |
